# Predictive value of serum tumor markers (carcinoembryonic antigen, neuron-specific enolase, and squamous cell carcinoma antigen in non-small cell lung cancer patients treated with programmed cell death protein 1 inhibitors

**DOI:** 10.5937/jomb0-54181

**Published:** 2025-06-13

**Authors:** Yang Wang, Danqing Li

**Affiliations:** 1 Hainan Cancer Hospital, Intensive Care Unit, Haikou, Hainan Province, China; 2 Xingtai People's Hospital, Department of Radiotherapy, Xingtai, Hebei Province, China

**Keywords:** carcinoembryonic antigen, neuron-specific enolase, squamous cell carcinoma antigen, non-small cell lung cancer, PD-1 inhibitors, adverse reactions, nomogram predictive model, multivariable logistic regression model, karcinoembrioni antigen, neuron-specifična enolaza, antigen karcinoma skvamoznih ćelija, nemikroćelijski karcinom pluća, PD-1 inhibitori, neželjene reakcije, prediktivni model nomograma, multivarijabilni logistički regresioni model

## Abstract

**Background:**

Adverse reactions (ARs) may occur in patients with advanced non-small cell lung cancer (ANSCLC) undergoing treatment with programmed cell death protein 1 (PD-1) inhibitors (PD-1Is). Establishing a risk assessment model can facilitate personalized treatment.

**Methods:**

Clinical data were collected from 215 ANSCLC patients treated with PD-1Is. Patients who experienced ARs were classified as the observation group (OG, 92 cases), while those who did not experience ARs were classified as the control group (CG, 123 cases). A multivariable logistic regression (LR) model was employed to analyze independent risk factors (RFs) associated with ARs, and R Studio software was utilized to create a nomogram predictive model.

**Results:**

The concordance index for the nomogram predictive model for ARs in ANSCLC patients treated with PD-1Is was 0.911. The threshold for predicting ARs using the nomogram was more significant than 0.25, providing a clinical net benefit superior to individual indicators such as smoking, tumour-node-metastasis (TNM) staging, neutrophil-to-lymphocyte ratio (NLR), systemic immune-inflammation index (SII), and prognostic nutritional index (PNI). The proportion of smokers in the OG was markedly superior to that in the CG (P<0.05).

**Conclusions:**

Smoking, TNM staging, and peripheral blood indicators such as NLR, SII, and PNI are independent RFs for the occurrence of ARs. The constructed nomogram predictive model demonstrates greater clinical utility than individual indicators, enhancing the accuracy of AR predictions.

## Abstract (sr)

**Uvod:**

Kod pacijenata sa uznapredovalim nemikroćelijskim karcinomom pluća koji se leče inhibitorima proteina programirane ćelijske smrti 1 (PD-1 inhibitori, PD-1I) mogu da se jave neželjene reakcije (NR). Uspostavljanje modela procene rizika može da olakša personalizovano lečenje.

**Metode:**

Prikupljeni su klinički podaci od 215 pacijenata sa uznapredovalim nemikroćelijskim karcinomom pluća (UNKP) lečenih PD-1 inhibitorima (PD-1I). Pacijenti kod kojih su se javile neželjene reakcije (NR) su klasifikovani u posmatranu grupu (PG, 92 slu~aja), dok su pacijenti bez NR klasifikovani u kontrolnu grupu (KG, 123 slu~aja). Za analizu nezavisnih faktora rizika (FR) povezanih sa NR korišćen je multivarijabilni logistički regresioni (LR) model, a za kreiranje prediktivnog modela nomograma korišćen je softver R Studio.

**Rezultati:**

Indeks podudarnosti prediktivnog modela nomograma za neželjene reakcije (NR) kod pacijenata sa uznapredovalim nemikroćelijskim karcinomom pluća lečenih PD-1 inhibitorima (PD-1I) je iznosio 0,911. Prag za predviđanje NR pomoću nomograma bio je značajniji od 0,25, pružajući kliničku neto korist veću od pojedinačnih pokazatelja, poput pušenja, TNM stadijuma (tumor-čvor-metastaze), odnosa neutrofila i limfocita (NLR), sistemskog indeksa imunološke inflamacije (SII) i prognostičkog nutritivnog indeksa (PNI). Udeo pušača u posmatranoj grupi je bio znatno veći nego u kontrolnoj grupi (KG) (P<0,05).

**Zaključak:**

Pušenje, TNM stadijum i periferni krvni indikatori poput NLR, SII i PNI su nezavisni faktori rizika (FR) za pojavu neželjenih reakcija (NR). Konstruisani prediktivni model nomograma pokazuje veću kliničku korisnost od pojedinačnih indikatora, povećavajući tačnost predviđanja NR.

## Introduction

Non-small cell lung cancer (NSCLC) accounts for approximately 85% of all lung cancer cases [1]. Traditional treatment modalities include surgery, radiation therapy, and chemotherapy; however, these approaches have advantages and disadvantages, and their applicability and efficacy can vary significantly among different patients [2] [3]. While surgery can offer a curative option for early-stage patients, its applicability is markedly reduced for those with advanced disease. Chemotherapy has shown some efficacy in prolonging survival and alleviating symptoms. Yet, it is often associated with significant adverse reactions (ARs), such as nausea, vomiting, and immunosuppression, which can severely impact the patient’s quality of life [4]. Although radiation therapy can effectively control local tumours, its efficacy in managing distant metastases is limited.

The emergence of immune checkpoint in hibitors, such as programmed cell death protein 1 (PD-1) inhibitors (PD-1Is), has brought new hope for NSCLC therapy. These agents enhance the immune response by lifting the suppression of tumour cells in the immune system, resulting in significant clinical efficacy [5] [6]. However, the application of PD-1Is is unsuitable for all patients, as their effectiveness and safety can vary due to individual differences. The primary advantages of PD-1Is lie in their specificity and durability in targeting the tumour immune microenvironment. Unlike traditional chemotherapy, the mechanism of action of PD-1Is allows the immune system to recognize and attack tumour cells actively, thereby achieving long-lasting anti-tumor effects [7].

Furthermore, the ARs associated with PD-1Is are generally milder, with common side effects including rash, fatigue, and endocrine disorders, resulting in better patient tolerance compared to the severe ARs often caused by chemotherapy. PD-1Is may also lead to immune-related ARs, such as pneumonia and hepatitis, which can be life-threatening in severe cases. This underscores the importance of predicting their ARs [8] [9] [10]. Although PD-1Is are effective in some patients, not all individuals benefit from this treatment. Research indicated that peripheral blood factors and pathological characteristics may be closely associated with the efficacy and ARs of immunotherapy [11]. For instance, lymphocyte counts in peripheral blood, levels of tumour markers, and cytokine profiles are believed to influence patient responses to PD-1Is [12] potentially. This finding highlights the importance of personalized treatment, advocating for developing more scientifically sound therapeutic strategies based on biomarker testing and comprehensive analysis before initiating PD-1Is therapy.

In the clinical practice of immunotherapy, predicting ARs not only aids in optimizing treatment regimens but also facilitates the development of more scientifically sound monitoring strategies. Through comprehensive analysis of various peripheral blood factors and pathological characteristics, this work aimed to identify specific biomarkers and subsequently establish an efficient predictive model for ARs, assisting physicians in conducting risk assessments before treatment. In summary, as immunotherapy rapidly advances, it becomes increasingly important to gain a deeper understanding of the mechanisms underlying ARs in NSCLC patients receiving PD-1Is. This work aimed to explore the association between peripheral blood factors, pathological characteristics, and ARs to PD-1Is therapy, with the expectation of providing new insights for clinical practice to enhance treatment efficacy and enhance patients’ quality of life.

## Materials and methods

### Research object

Data were collected from the hospital’s electronic medical records system for 215 patients with advanced NSCLC (ANSCLC) who received PD-1Is at Hainan Cancer Hospital between February 2022 and May 2024. Before conducting this study, we obtained approval from the ethics committee of Hainan Cancer Hospital. All patient data were anonymized, and no information that could potentially identify individual patients was involved in the research process.

Inclusion criteria included patients aged 18 years; patients’ electronic medical records must consist of clinical data before and after treatment, including information on peripheral blood factors and pathological characteristics; diagnosis of primary ANSCLC confirmed by histopathology; patients received PD-1Is treatment and had relevant follow-up records; patients must have a performance status of 0–2 on the Eastern Cooperative Oncology Group (ECOG) scale; patients must have measurable disease as defined by Response Evaluation Criteria in Solid Tumors (RECIST) version 1.1; patients must have adequate bone marrow, hepatic, and renal function as determined by laboratory tests; and patients must have provided informed consent for the use of their medical records in the study [13] [14] [15].

Exclusion criteria: i.) Patients with severe comorbidities (e.g., severe heart disease, hepatic or renal failure) during treatment; ii.) Patients with a history of other malignancies (excluding skin cancer); iii.) Patients who did not undergo effective follow-up after receiving PD-1Is treatment and lacked relevant efficacy or AR data; iv.) Patients receiving other immuno therapies or systemic therapy concurrently with PD-1I treatment; v.) Patients with infections within one week before treatment.

### Data collection

The clinical data of the patients included the following:

i. Demographic information: gender, age, smoking status, alcohol consumption, hypertension, hyperlipidemia, and body mass index (BMI).

ii. Pathological type: adenocarcinoma, squamous cell carcinoma, sarcoma, and other types.

iii. Clinical tumour-node-metastasis (TNM) staging: patient pathological staging was classified according to the 8th edition of the *International Union Against Cancer* tumour staging standards.

iv. Peripheral blood factors:

– Serum tumour markers: carcinoembryonic antigen (CEA), neuron-specific enolase (NSE), and squamous cell carcinoma antigen (SCC).

– Complete blood count indicators: blood platelet (PLT) count, neutrophil (NEU) count, and C-reactive protein (CRP) levels.

– Biochemical indicators: albumin (ALB), blood urea nitrogen (BUN), and serum creatinine (Scr).

– Neutrophil-to-lymphocyte ratio (NLR): calculated as the absolute neutrophil count divided by the absolute lymphocyte count.

– Systemic immune-inflammation index (SII) and prognostic nutritional index (PNI).

v. ARs related to PD-1Is treatment included skin reactions (such as rash, itching, and dry skin), endocrine reactions (thyroid dysfunction, pituitary insufficiency, and adrenal insufficiency), pulmonary reactions (such as dyspnea and cough), hepatic reactions (liver function impairment), allergic reactions (drug allergies and injection site reactions), infections, and non-immune-related side effects (such as infusion reactions and venous thrombosis). Among these, skin reactions, endocrine reactions, pulmonary reactions, and hepatic reactions were categorized as ARs. Patients who experienced these reactions were classified as the observation group (OG, 92 cases), while those without ARs were classified as the control group (CG, 123 cases).

### Statistical methodologies

Data processing was conducted using SPSS version 22.0. Normally distributed continuous data were denoted as mean ± standard deviation (x̄±s), while categorical data as frequency and percentage (%). The Mann-Whitney U test compared groups for non-normally distributed continuous data, whereas one-way ANOVA was employed for normally distributed data. Categorical data were compared employing the chi-square test. A multivariable logistic regression (LR) model analyzed independent risk factors (RFs) associated with ARs in patients. Using R Studio (embedded R version 3.6.3), all patient data were randomly rolled into training and testing sets at a ratio of 7:3, with the training set constructing the predictive model and the testing set conducting model validation. The model’s predictive value was calculated using the area under the receiver operating characteristic curve (AUC-ROC) to assess its discriminatory power. A nomogram was created to illustrate the impact of different predictive factors on patient outcomes. A two-tailed test was considered statistically significant at *P*<0.05.

## Results

### Demographic data

In the study, patients who experienced adverse reactions (ARs) were classified as the Observation Group (OG, n=92), while those without ARs were classified as the Control Group (CG, n=123). The gender ratio (52.2% male in OG vs 51.2% male in CG), age (55.3±10.2 years in OG vs 54.8±9.9 years in CG), alcohol consumption (29.3% in OG vs 27.6% in CG), hypertension (34.8% in OG vs 31.7% in CG), hyperlipidemia (25.0% in OG vs 22.8% in CG), and BMI (26.5±3.2 kg/m^2^ in OG vs. 26.8±3.1 kg/m^2^ in CG) exhibited negligible differences between the groups (P>0.05 for all). However, the proportion of smokers was significantly higher in the OG (55.4%) compared to the CG (35.0%), with a P-value of 0.041.

### Pathological types

In Table 1, the OG included 48 cases of adenocarcinoma (52.17%), 29 cases of squamous cell carcinoma (31.52%), 11 cases of sarcoma (11.96%), and 4 cases of other types (4.35%). In the CG, there were 62 cases of adenocarcinoma (50.41%), 38 cases of squamous cell carcinoma (30.89%), 16 cases of sarcoma (13.01%), and 7 cases of other types (4.88%). There were inconsiderable differences in the proportions of adenocarcinoma, squamous cell carcinoma, sarcoma, and other types when comparing the OG and CG (*P*>0.05).

**Table 1:** Demographic and Clinical Characteristics of the Observation Group (OG) and Control Group (CG).

Variable	Observation Group (OG, n=92)	Control Group (CG, n=123)	P-value
Gender (M/F)	48 (52.2%) / 44 (47.8%)	63 (51.2%) / 60 (48.8%)	0.873
Age (years)	55.3±10.2	54.8±9.9	0.789
Alcohol Consumption	27 (29.3%)	34 (27.6%)	0.845
Hypertension	32 (34.8%)	39 (31.7%)	0.712
Hyperlipidemia	23 (25.0%)	28 (22.8%)	0.765
BMI (kg/m^2^)	26.5±3.2	26.8±3.1	0.634
Smoking Status	51 (55.4%)	43 (35.0%)	0.041

In Table 2, the OG included 41 cases of stage III (44.57%) and 51 cases of stage IV (55.43%). In the CG, there were 84 cases of stage III (68.29%) and 39 cases of stage IV (31.71%). The proportion of stage III patients in the OG was lower than that in the CG, while the proportion of stage IV patients was higher in the OG, with substantial differences observed (*P*<0.05).

**Table 2:** Distribution of Pathological Types and Stages in the Observation Group (OG) and Control Group (CG).

Category	Observation Group (OG) (n=92)	Control Group (CG) (n=123)	P-value
Pathological Type			
Adenocarcinoma	48 (52.17%)	62 (50.41%)	0.789
Squamous Cell Carcinoma	29 (31.52%)	38 (30.89%)	0.876
Sarcoma	11 (11.96%)	16 (13.01%)	0.745
Other Types	4 (4.35%)	7 (4.88%)	0.892
Stage			
Stage III	41 (44.57%)	84 (68.29%)	<0.05
Stage IV	51 (55.43%)	39 (31.71%)	<0.05

**Figure 1:**
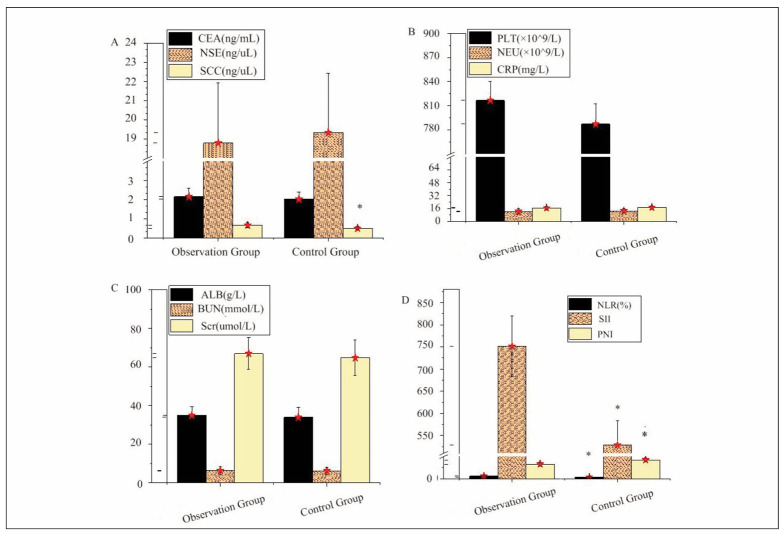
Comparison of peripheral blood factors between OG and CG. (A: CEA, NSE, SCC; B: PLT, NEU, CRP; C: ALB, BUN, Scr; D: NLR, SII, PNI).

In Figure 1, the serum tumour markers CEA, NSE, SCC, complete blood count indicators PLT, NEU, CRP, and biochemical indicators ALB, BUN, and Scr differed slightly between the OG and CG (*P*>0.05). However, SCC, NLR, and SII in the OG were drastically superior to those in the CG, while the PNI level was greatly lower in the OG, with drastic differences observed (*P*<0.05).

### Univariate LR analysis of ARs in patients

Based on the inter-group comparison results, smoking status, TNM staging, and peripheral blood factors SCC, NLR, SII, and PNI were included as independent variables, while the occurrence of ARs in patients served as the dependent variable for multivariable LR analysis (Table 3). The impacts of smoking, TNM staging, NLR, SII, and PNI levels on the occurrence of ARs were statistically notable (*P*<0.05). In contrast, the effect of SCC on ARs showed a neglectable difference (*P*>0.05).

**Table 3:** Univariate LR analysis of ARs in patients.

Variable	Regression coefficient (β)	OR	95% CI	P
Smoking	0.916	2.57	(1.20, 5.00)	<0.05
TNM staging	1.098	3.94	(1.50, 6.00)	<0.001
NLR	0.587	3.07	(1.10, 4.40)	<0.001
SII	0.788	2.98	(1.30, 4.00)	<0.05
PNI	-0.693	3.38	(1.10, 4.40)	<0.05
SCC	0.095	1.24	(0.70, 1.70)	>0.05

### Multivariate LR analysis of ARs in patients

Based on the results of the univariate LR analysis, smoking status, TNM staging, and peripheral blood factors NLR, SII, and PNI were included as independent variables, while the occurrence of ARs in patients served as the dependent variable for multivariate LR analysis (Table 4). The impacts of smoking, TNM staging, NLR, SII, and PNI levels on the occurrence of ARs were statistically considerable (*P*<0.05).

**Table 4:** Multivariate LR analysis of ARs in patients.

Variable	Regression coefficient (β)	OR	95% CI	P
Smoking	0.845	2.33	(1.15, 4.71)	<0.05
TNM staging	1.245	3.47	(1.68, 7.15)	<0.001
NLR	0.684	1.93	(1.12, 3.34)	<0.05
SII	0.912	2.49	(1.20, 5.19)	<0.001
PNI	-0.578	0.56	(0.34, 0.90)	<0.05

### Construction of the AR prediction model

**Figure 2:**
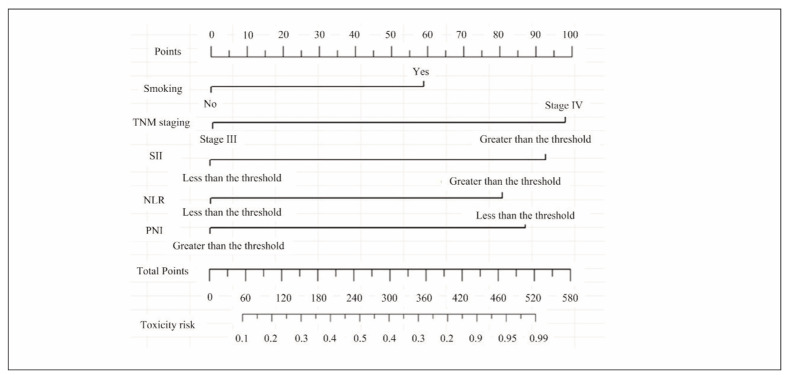
Nomogram prediction model for ARs in patients with ANSCLC undergoing PD-1I therapy.

Using smoking status, TNM staging, NLR, SII, and PNI as predictive factors, a nomogram prediction model was developed to assess the risk of ARs in patients with ANSCLC undergoing PD-1I therapy (Figure 2).

### Calibration and decision curve of the nomogram prediction model

**Figure 3:**
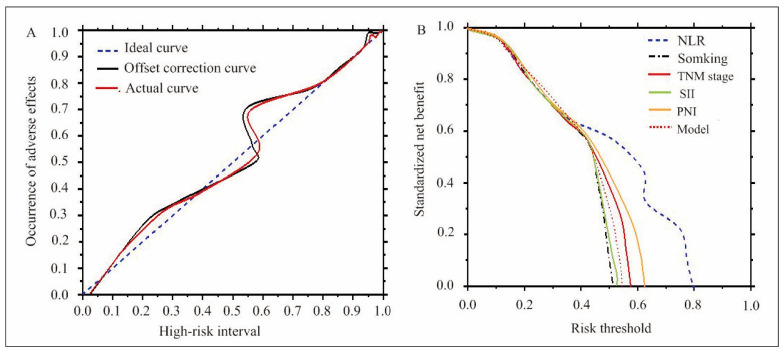
Calibration and decision curves of the nomogram prediction model. (A: calibration curve; B: decision curve).

In Figure 3, the concordance index of the nomogram prediction model for predicting ARs in patients with ANSCLC undergoing PD-1I therapy was 0.911, indicating a calibration curve that closely aligns with the actual curve. Figure 6B illustrates that the threshold for the nomogram prediction model’s AR prediction was more significant than 0.25, and it provided a clinical net benefit that surpasses that of individual indicators such as smoking status, TNM staging, NLR, SII, and PNI.

## Discussion

In this study, patients undergoing PD-1 inhibitor therapy for advanced non-small cell lung cancer (ANSCLC) were classified into an Observation Group (OG, n=92) with adverse reactions (ARs) and a Control Group (CG, n=123) without ARs. Demo graphic and clinical characteristics were similar between groups, except for a significantly higher proportion of smokers in the OG (55.4% vs. 35.0%, P=0.041). Pathological types were not significantly different, but the OG had a higher proportion of stage IV patients (55.43% vs. 31.71%, P<0.05). Peripheral blood factors, including SCC, NLR, SII, and PNI, showed significant differences, with higher SCC, NLR, and SII levels and lower PNI levels in the OG (P<0.05). Univariate and multivariate logistic regression analyses identified smoking, TNM staging, NLR, SII, and PNI as significant predictors of ARs (P<0.05). A nomogram prediction model incorporating these factors was developed, demonstrating a high concordance index (0.911) and providing a clinical net benefit that surpasses individual indicators. In contrast, a study by Guo et al. [13] found that PD-1/PD-L1 inhibitors were associated with a lower incidence of irAEs, including pneumonitis, in patients with NSCLC. Another study by Zhou et al. [14] reported that PD-1 inhibitors were associated with a higher incidence of irAEs, including pneumonitis, than PD-L1 inhibitors.

Our study also found that smoking, TNM staging, NLR, SII, and PNI were significant predictors of irAEs, consistent with a study by Liang et al. [15]. However, our study found that the incidence of irAEs was higher in patients with stage IV disease, which contrasts the findings of a survey by Ladjevardi et al. [16]. Overall, our study suggests that PD-1 inhibitors are associated with a higher incidence of irAEs in patients with NSCLC, particularly in those with stage IV disease, and that smoking, TNM staging, NLR, SII, and PNI are significant predictors of irAEs.

Studies have shown that irAEs are associated with the efficacy of immune checkpoint inhibitors in patients with advanced non-small-cell lung cancer (NSCLC). A meta-analysis of 35 studies covering 8435 patients with advanced NSCLC found that irAEs were associated with improved objective response rate, overall survival, and progression-free survival [17].

Another study found that the incidence of irAEs was higher in patients with NSCLC who received PD-1/PD-L1 inhibitors plus chemotherapy compared to those who received PD-1/PD-L1 inhibitors alone [18]. However, adding chemotherapy to PD-1/PD-L1 inhibitors may reduce the incidence of irAEs [19].

We found that smoking history has been identified as a risk factor for immune-related adverse events (irAEs) in patients with NSCLC treated with immune checkpoint inhibitors (ICIs). This is confirmed in multiple studies. A systematic review and meta-analysis found that smoking history was associated with an increased risk of pneumonitis, a severe irAE, in patients with NSCLC treated with ICIs [20]. Another study found that smoking history was a risk factor for irAEs in patients with NSCLC treated with ICIs, along with pre-existing interstitial lung disease and male sex [21]. A review of the literature on pulmonary adverse events following ICIs also noted that smoking history was a risk factor for pneumonitis in patients with NSCLC [22].

A study published by Dupont et al. [23] found that the incidence of irAEs was higher in patients with stage IV NSCLC compared to those with earlier stages. As well as another study by Liu et al. [24] found that the incidence of thyroid dysfunction was higher in patients with stage III-IV NSCLC compared to those with stage I-II. These confirmed our study findings.

The comparison of demographic characteristics such as M/F ratio, age, alcohol consumption, HTN, hyperlipidemia, and BMI between the OG and CG showed no significant differences. However, the smoking rate was higher in the OG, suggesting smoking may be an independent RF for ARs in PD-1I therapy. Pathological types (AD, SCC, sarcoma, other) were similar in both groups, indicating pathological type may not influence ARs. Serum tumour markers (CEA, NSE, SCC), blood routine indicators (PLT, NEU, CRP) and biochemical indicators (ALB, BUN, Scr) showed no significant differences. The OG had higher SCC, NLR, and SII levels and a lower PNI level. Elevated SCC may indicate higher tumour activity and NLR and SII, suggesting a more pronounced inflammatory response. This heightened inflammatory response may be a contributing factor to the increased incidence of ARs in the OG. The significant decrease in PNI is typically associated with malnutrition or a compromised immune status. The notable reduction in PNI in the OG may indicate a poorer overall health condition of the patients, potentially affecting their tolerance to treatment and thereby increasing the risk of ARs [25]. This finding aligns with the results from Gong et al. [26], which explored prognostic factors in lung cancer patients, suggesting that levels of SCC, NLR, SII, and PNI may serve as important indicators of patients’ tolerance to PD-1I treatment and their overall prognosis.

## Conclusion

This study explored the predictive factors for ARs in patients with ANSCLC undergoing treatment with PD-1Is. By analyzing multiple factors, including smoking, TNM staging, and peripheral blood indicators such as NLR, SII, and PNI, it was found that these variables significantly contributed to the prediction of ARs, suggesting that they may serve as independent RFs. Although SCC did not demonstrate statistical significance, its relationship with other factors warrants further investigation. The constructed nomogram prediction model exhibited strong predictive capability, providing physicians with personalized risk assessments to optimize patient management. Furthermore, the calibration curve and decision curve analyses indicated that the nomogram model, which integrates multiple factors, holds greater clinical value compared to single indicators, enhancing the accuracy of predicting ARs.

Although this study provides a valuable predictive model for ARs in patients with ANSCLC undergoing PD-1I treatment, it does have several limitations. First, the research primarily relies on electronic medical record data, which may be affected by incomplete or inconsistent data recording, potentially limiting the accuracy of the results. Additionally, the study did not investigate the dynamic changes of potential biomarkers related to ARs, such as alterations in the tumour microenvironment during treatment, which could impact the predictive capability of the model. Future research should focus on exploring multidimensional biomarkers, including circulating tumour DNA, immune cell phenotypes, and their activity, to gain a deeper understanding of their roles in ARs. Furthermore, employing advanced data analysis methods like machine learning could help construct more complex predictive models for effective personalized treatment. Considering subjective factors like patients’ psychological states and quality of life may also provide new perspectives for enhancing treatment efficacy and patient satisfaction. This approach will promote more comprehensive and personalized cancer management strategies, ultimately leading to better clinical outcomes.

## Dodatak

### Author contributions

All authors contributed to the study’s conception and design. Yang Wang and Danqing Li performed material preparation, data collection and analysis. Yang Wang, and Danqing Liand, wrote the first draft of the manuscript; all authors commented on previous versions of the manuscript. All authors read and approved the final manuscript.

### Ethical standards

The authors have no relevant financial or non-financial interests to disclose.

### Ethical approval statement

This retrospective chart review study involving human participants followed the institutional and national research committee’s ethical standards, the 1964 Helsinki Declaration, and its later amendments or comparable ethical standards. The Human Investigation Committee (IRB) of Hainan Cancer Hospital approved this study (Approval number: HCH021).

### Informed consent

The authors affirm that human research participants provided informed consent to publish the images in Figure(s) Figure 1A, Figure 1B and Figure 1C. The participant has consented to the submission of the case report to the journal. Patients signed informed consent regarding publishing their data and photographs.

### Availability of data and materials

The datasets generated during and/or analyzed during the current study are available from the corresponding author upon reasonable request.

### Acknowledgements

None.

### Funding source

The study was supported by Hainan Cancer Hospital by code 3547E/2517.

### Conflict of interest statement

All the authors declare that they have no conflict of interest in this work.
